# Disparities and trends in pulmonary embolism mortality with and without obesity: a nationwide US analysis

**DOI:** 10.1016/j.rpth.2025.103240

**Published:** 2025-10-30

**Authors:** Aman Goyal, Humza Saeed, Samia Aziz Sulaiman, Muhammad Khubaib Arshad, Kevin Michael Alexander, Sripal Bangalore, Liana K. Billings, Alfonso J. Tafur, Manan Pareek, Gregory Piazza, Arman Qamar

**Affiliations:** 1 Department of Internal Medicine, Cleveland Clinic Foundation, Cleveland, Ohio, USA; 2 Department of Internal Medicine, Rawalpindi Medical University, Rawalpindi, Pakistan; 3 Department of Internal Medicine, University of Jordan, School of Medicine, Jordan; 4 Division of Cardiovascular Medicine, Stanford University School of Medicine, Stanford, California, USA; 5 Division of Cardiology, Department of Medicine, New York University Grossman School of Medicine, New York, New York, USA; 6 Department of Medicine, Endeavor Health/NorthShore, Skokie, Illinois, USA; 7 Department of Medicine, Pritzker School of Medicine, University of Chicago, Chicago, Illinois, USA; 8 Department of Vascular Medicine, Endeavor Health, Pritzker School of Medicine, University of Chicago, Chicago, Illinois, USA; 9 Department of Cardiology, Copenhagen University Hospital—Rigshospitalet, Copenhagen, Denmark; 10 Division of Cardiovascular Medicine, Brigham and Women’s Hospital, Boston, Massachusetts, USA; 11 Division of Interventional Cardiology and Vascular Medicine, NorthShore University Health System, Pritzker School of Medicine, University of Chicago, Chicago, Illinois, USA

**Keywords:** disparity, epidemiology, mortality, obesity, pulmonary embolism, risk factor

## Abstract

**Background:**

Obesity increases the risk of pulmonary embolism (PE) through multiple mechanisms.

**Objectives:**

This study examined mortality trends in patients with coexisting PE and obesity.

**Methods:**

We analyzed the Centers for Disease Control and Prevention Wide-ranging Online Data for Epidemiologic Research database for individuals aged 25 years and older who died between 1999 and 2020 from concurrent PE and obesity. Temporal trends and age-adjusted mortality rates (AAMRs) were assessed using Joinpoint regression software. Annual percent changes (APCs), average annual percentage change differences, and 95% CIs were calculated, with statistical significance set at *P* < .05.

**Results:**

From 1999 to 2020, the AAMR for PE with obesity increased from 5.1 (95% CI, 4.7-5.4) to 13.9 (95% CI, 13.4-14.4) per 1,000,000. The AAMR rose significantly from 1999 to 2018 (APC, 3.45; 95% CI, 2.65-4.01; *P* = .005), followed by a sharper increase from 2018 to 2020 (APC, 16.28; 95% CI, 6.24-21.22; *P* < .001). Women consistently had higher AAMRs than men (8.8; 95% CI, 8.6-8.9; vs 6.5; 95% CI, 6.4-6.6). Among age groups, middle-aged adults had the highest AAMR (10; 95% CI, 9.8-10.1), while among the ethnoracial groups, non-Hispanic Black individuals had the highest AAMR (16.8; 95% CI, 16.5-17.2). Residents of nonmetropolitan areas had higher AAMRs than those in metropolitan areas (8.9; 95% CI, 8.7-9.1; vs 7.5; 95% CI, 7.4-7.6). The increase in AAMR was significantly greater for PE with obesity compared with PE alone (average annual percentage change difference, 3.61; 95% CI, 2.91-4.32; *P* < .001).

**Conclusions:**

The analysis reveals a significant rise in mortality from concurrent PE and obesity, with higher rates observed in women, middle-aged adults, non-Hispanic Black individuals, and residents of nonmetropolitan areas. These findings highlight the need for targeted interventions in these high-risk groups.

## Essentials

• Data were analyzed to assess mortality trends in pulmonary embolism (PE) with and without obesity.
• Concurrent obesity and PE were associated with higher mortality rates compared with PE alone.
• Mortality rates for PE with obesity increased significantly, particularly from 2018 to 2020.
• Women, middle-aged adults, and Black individuals had higher mortality than their counterparts.

## Graphical abstract

**Figure:**
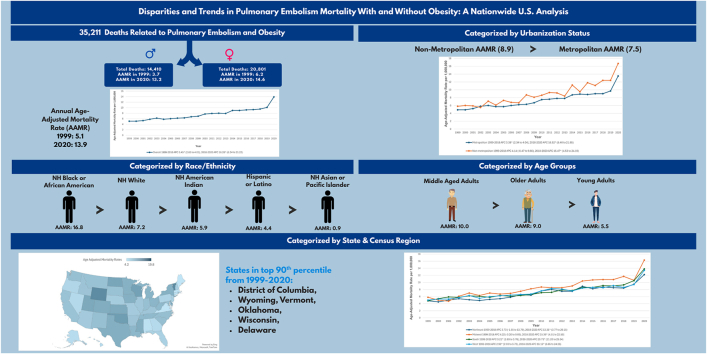

## 1 Introduction

Pulmonary embolism (PE) represents the most severe manifestation of venous thromboembolism and remains a major cause of morbidity and mortality worldwide [1]. It is a prevalent cardiovascular disorder that contributes significantly to the overall global burden of cardiovascular disease [2]. The annual incidence rate of PE varies widely across regions, ranging from 0.039 per 1000 people in Hong Kong to 1.15 per 1000 in the United States [3]. Despite these variations, the global incidence of PE has been on an upward trend in recent years [1]. Previous studies have explored trends in PE incidence and mortality, with Huisman et al. [1] demonstrating that PE incidence has been increasing in both sexes [1]. Obesity has become increasingly concerning over recent years. It is projected that by 2035, the global prevalence of overweight and obesity will reach 51%, and by 2030, 78% of adults in the United States are expected to be overweight or obese [4]. Moreover, studies have shown that obese individuals are more likely to be hospitalized for other medical conditions that could increase their risk for PE, further raising their likelihood of complications [5].

Trends in obesity prevalence indicate higher rates among women, socioeconomically disadvantaged ethnoracial groups, and individuals with lower educational attainment, which further complicates its association with PE-related mortality [4,5]. Research has demonstrated that the incidence of PE is greater in obese women compared with obese men, suggesting potential sex-specific mechanisms through which obesity influences PE [5]. While prior studies have examined mortality trends for obesity and PE separately [6,7], limited research exists on PE-related mortality trends in individuals with concurrent obesity. Moreover, the rise in PE incidence may be attributed to several factors—including greater awareness and advances in diagnostic imaging that have improved detection rates—the increasing prevalence of obesity, a well-established risk factor for PE, is particularly significant in the contemporary era [1,3]. With evolving and increasingly effective therapeutic advances in obesity management, tracking mortality trends related to obesity and PE could help elucidate the burden of mortality from these conditions over time and support the development of improved monitoring guidelines and potentially more aggressive management strategies. Therefore, our study aimed to analyze PE-related mortality trends in individuals with concurrent obesity from 1999 to 2020. By comparing these trends to those observed in individuals with PE or obesity alone, we aimed to better understand the potential interplay between these conditions and how various factors—such as age group, sex, ethnoracial background, and urbanization status—influence these trends.

## 2 Methods

### 2.1 Study setting and population

We obtained mortality data from the Centers for Disease Control and Prevention Wide-ranging Online Data for Epidemiologic Research (CDC WONDER) database [8] and analyzed mortality rates among adults with both PE and obesity from 1999 to 2020. Specifically, we used the Multiple Cause of Death Public Use Record database to identify cases where PE and obesity were both listed as contributing causes on US death certificates [9]. This approach has been applied in several previous CDC WONDER–based studies [[10], [11], [12]]. We identified PE and obesity cases using the following International Classification of Diseases, 10th Revision (ICD-10) codes: I26 for PE and E66 for obesity [13]. Since we utilized deidentified public-use data provided by the government, our study was exempt from institutional review board approval, following the guidelines of Strengthening the Reporting of Observational Studies in Epidemiology (STROBE) [14].

### 2.2 Data abstraction

Our demographic variables were defined by population size, age distribution, sex composition, ethnoracial background, geographic location, and urbanization level, covering the period from 1999 to 2020. The locations of death included inpatient facilities, outpatient clinics, emergency departments, sudden death cases, residences, hospices/nursing homes, long-term care facilities, and unspecified locations. Ethnoracial categories were classified as Hispanic (Latino), non-Hispanic (NH) White, NH Black, NH American Indian/Alaskan Native, and NH Asian. These classifications are consistent with previous analyses using the CDC WONDER database and adhere to the US Office of Budget and Management Guidelines as recorded on death certificates [9].

Patients were segmented into 3 age groups: young adults (25-44 years), middle-aged adults (45-64 years), and older adults (≥65 years), in line with age distribution criteria used in prior CDC WONDER studies [15]. Geographic stratification followed the Urban-Rural Classification Scheme from the National Center for Health Statistics, dividing counties into urban (large metropolitan areas with populations over 1 million), medium/small metropolitan areas (populations between 50,000 and 999,999), and rural/nonmetropolitan areas (populations under 50,000). Additionally, the United States was divided into 4 regions—Northeast, Midwest, South, and West—according to US Census Bureau classifications [16]. We also analyzed the trends for individual PE and obesity-related AAMRs to make comparisons with the combined mortality.

### 2.3 Statistical analysis

We examined patterns related to sex, race, age, urbanization, and census regions by calculating the age-adjusted mortality rates (AAMRs) per 1,000,000 people, using the 2000 US population as the baseline for AAMR standardization [17]. Annual population estimates used as denominators were obtained from the CDC WONDER–bridged race population estimates, based on data from the US Census Bureau and National Center for Health Statistics. Temporal shifts in mortality rates were evaluated through the Joinpoint Regression Program (version 5.0.2; National Cancer Institute) [18], applying log-linear regression models to assess trends over time. Joinpoint regression was used to identify inflection points in AAMR trends for patients with coexisting PE and obesity from 1999 to 2020, as outlined in published guidelines. For datasets with 17 to 21 time points, the recommendation is to detect up to 3 inflection points. However, given our 22-year study span, we configured the software to identify up to 4 joinpoints where significant changes in trends occurred. Fewer joinpoints could be detected if fewer points sufficiently captured trend variability. The Grid Search method (2,2,0), permutation test, and parametric approach were used to calculate annual percent change (APC) and 95% CIs. An APC was considered increasing or decreasing if the slope for the mortality trend differed significantly from 0, based on 2-tailed *t*-tests. In addition, Joinpoint software was used to calculate the average annual percentage change from 1999 to 2020 for combined PE with concurrent obesity, PE alone, and obesity alone. To assess differences in trends between these categories, we conducted nonparallel pairwise comparisons using the average annual percentage change difference (AAPCD). Statistical significance was set at a 2-sided *P* ≤ .05.

## 3 Results

### 3.1 Overall demographic trends in mortality

**Figure 1:**
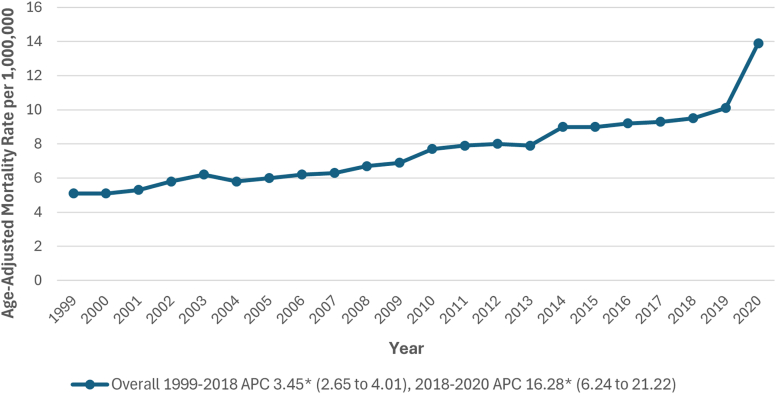
Overall pulmonary embolism and obesity-related age-adjusted mortality rates per 1,000,000 in adults in the United States, 1999 to 2020. ∗The annual percentage change (APC) was significantly different from zero at α = 0.05.

Between 1999 and 2020, 35,211 deaths were related to both PE and obesity in individuals aged ≥25 years. The AAMR increased from 5.1 (95% CI, 4.7-5.4) in 1999 to 13.9 (95% CI, 13.4-14.4) per 1,000,000 individuals in 2020. From 1999 to 2018, the AAMR showed a significant rise (APC, 3.45; 95% CI, 2.65 to 4.01; *P* = .005), followed by a sharper rise from 2018 to 2020 (APC, 16.28; 95% CI, 6.24 to 21.22; *P* < .001) (Figure 1 and Supplementary Tables 1 and 2).

### 3.2 Sex stratification

**Figure 2:**
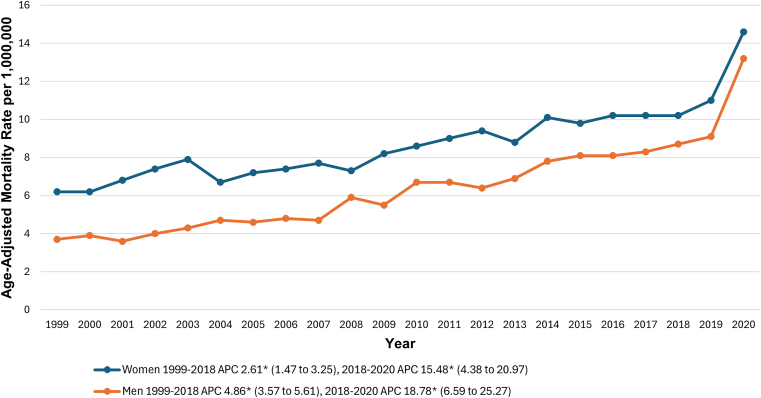
Pulmonary embolism and obesity -related age-adjusted mortality rates per 1,000,000, stratified by sex in adults in the United States, 1999 to 2020. ∗The annual percentage change (APC) was significantly different from zero at α = 0.05.

During the study period from 1999 to 2020, women consistently had higher AAMRs than men (8.8; 95% CI, 8.6-8.9; vs 6.5; 95% CI, 6.4-6.6) per 1,000,000, respectively. Both sexes exhibited similar trends, with a significant increase from 1999 to 2018 (men’s APC, 4.86; 95% CI, 3.57-5.61; *P* = .007; women’s APC, 2.61; 95% CI, 1.47-3.25; *P* = .015). This was followed by a sharp increase between 2018 and 2020 (men’s APC, 18.78; 95% CI, 6.59-25.27; *P* < .001; women’s APC, 15.48; 95% CI, 4.38-20.97; *P* < .001) (Figure 2 and Supplementary Tables 2 and 3).

### 3.3 Age group stratification

**Figure 3:**
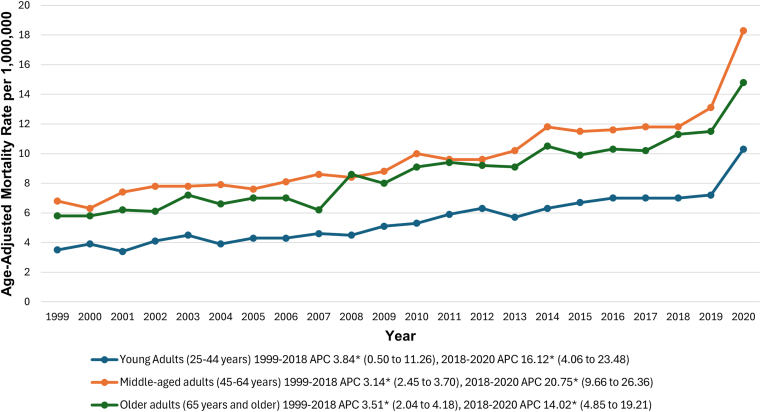
Pulmonary embolism and obesity-related age-adjusted mortality rates per 1,000,000, stratified by age groups in adults in the United States, 1999 to 2020. ∗The annual percentage change (APC) was significantly different from zero at α = 0.05.

AAMRs were highest among middle-aged adults at 10.0 (95% CI, 9.8-10.1) per 1,000,000, followed by older adults (9.0; 95% CI, 8.8-9.1) and young adults (5.5; 95% CI, 5.3-5.6). All 3 age groups—young adults, middle-aged adults, and older adults—exhibited similar trends, with a significant increase from 1999 to 2018, followed by an inflection point in 2018 and a steeper upward trend from 2018 to 2020. Further details are provided in Figure 3 and Supplementary Tables 2 and 4 (*P* < .05 for all APCs).

### 3.4 Ethnoracial stratification

**Figure 4:**
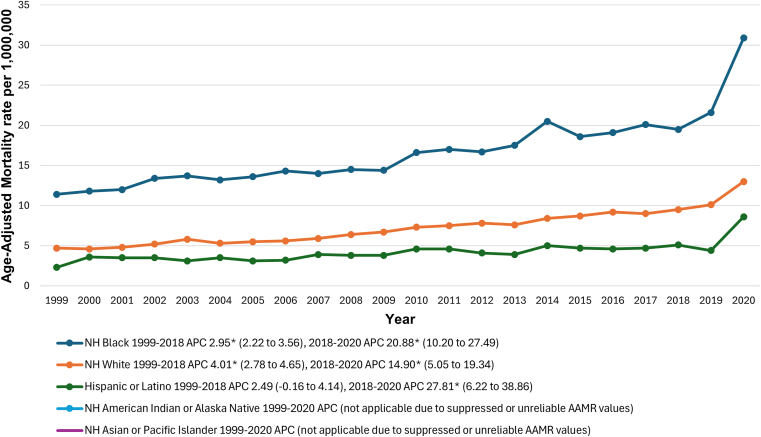
Pulmonary embolism and obesity-related age-adjusted mortality rates (AAMRs) per 1,000,000, stratified by ethnoracial groups in adults in the United States, 1999 to 2020. ∗The annual percentage change (APC) was significantly different from zero at α = 0.05.

AAMRs were the highest among NH Black individuals at 16.8 (95% CI, 16.5-17.2) per 1,000,000, followed by those among NH White (7.2; 95% CI, 7.1-7.3), NH American Indian or Alaska Native (5.9; 95% CI, 5.0-6.7), Hispanic (4.4; 95% CI, 4.2-4.6), and NH Asian or Pacific Islander (0.9; 95% CI, 0.8-1.0) populations. Due to low numbers and unreliable values, trends were not assessed for NH American Indian and NH Asian groups. In NH Black and NH White individuals, AAMRs increased significantly from 1999 to 2018 (NH Black adults’ APC, 2.95; 95% CI, 2.22-3.56; *P* = .002; NH White adults’ APC, 4.01; 95% CI, 2.78-4.65; *P* = .009), while Hispanic individuals did not show a significant increase during the same period (APC, 2.49; 95% CI, −0.16 to 4.14; *P* = .057). From 2018 to 2020, however, all 3 ethnoracial groups showed steep, upward trends (NH Black adults’ APC, 20.88; 95% CI, 10.20-27.49; *P* < .001; NH White adults’ APC, 14.90; 95% CI, 5.05-19.34; *P* < .001; Hispanic adults’ APC, 27.81; 95% CI, 6.22-38.86; *P* < .001) (Figure 4 and Supplementary Tables 2 and 5).

### 3.5 State-wise distribution

**Figure 5:**
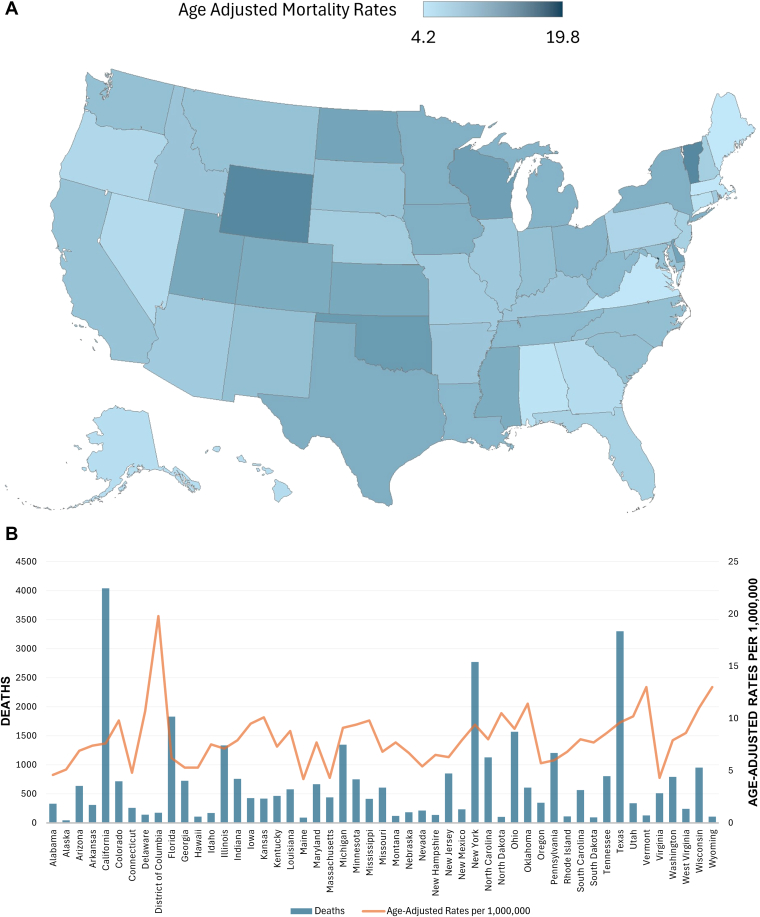
Pulmonary embolism and obesity-related age-adjusted mortality rates (AAMRs) per 1,000,000, stratified by state in adults in the United States, 1999 to 2020. (A) Map displaying AAMRs by state, using a continuous color gradient scale ranging from 4.2 to 19.8, with lighter shades indicating lower mortality rates and darker shades indicating higher mortality rates. (B) Bar chart illustrating the total number of deaths and corresponding AAMRs by state. This figure highlights geographic variation in both absolute mortality burden and age-adjusted rates across the United States.

AAMRs varied significantly by state, ranging from 4.2 (95% CI, 3.4-5.2) per 1,000,0000 in Maine to 19.8 (95% CI, 16.8-22.7) in the District of Columbia. States in the top 90th percentile (District of Columbia, Wyoming, Vermont, Oklahoma, Wisconsin, and Delaware) had AAMRs approximately 2 to 3 times higher than those in the bottom 10th percentile (Alaska, Connecticut, Alabama, Virginia, Massachusetts, and Maine) (Figure 5 and Supplementary Table 6).

### 3.6 Census region

**Figure 6:**
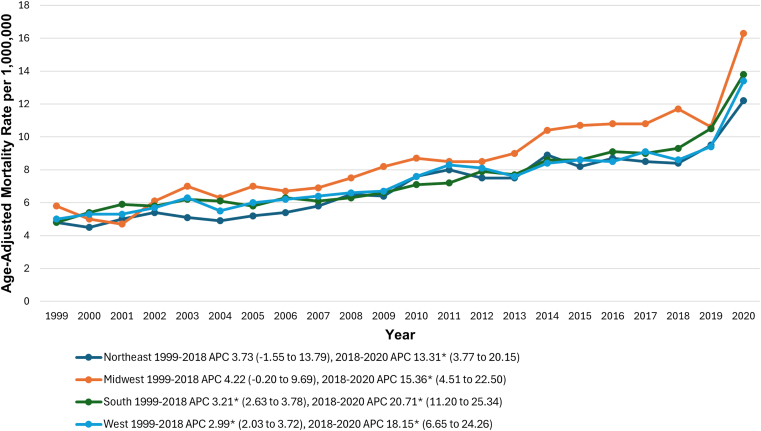
Pulmonary embolism and obesity-related age-adjusted mortality rates per 1,000,000, stratified by census regions in adults in the United States, 1999 to 2020. ∗The annual percentage change (APC) was significantly different from zero at α = 0.05.

Across census regions, the Midwestern region had the highest AAMR at 8.6 (95% CI, 8.4-8.8) per 1,000,000, followed by the Southern (7.6; 95% CI, 7.5-7.7) and Western regions (7.6; 95% CI, 7.4-7.7), with the Northeastern region having the lowest (7.0; 95% CI, 6.8-7.2). Further details are depicted in Figure 6 and Supplementary Tables 2 and 7.

### 3.7 Urbanization

**Figure 7:**
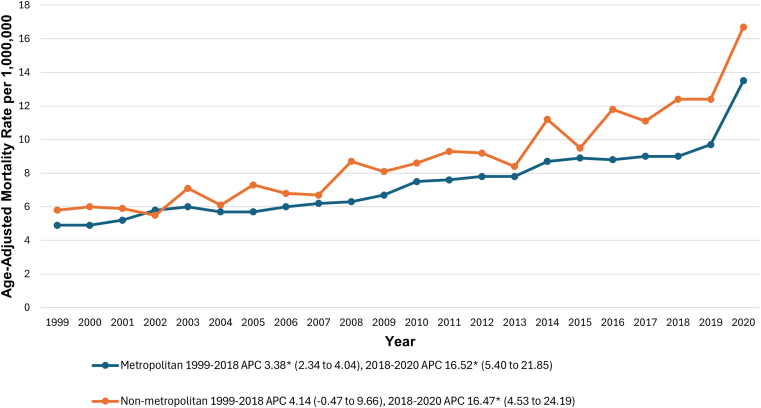
Pulmonary embolism and obesity-related age-adjusted mortality rates per 1,000,000 in adults in the metropolitan and nonmetropolitan areas in the United States, 1999 to 2020. ∗The annual percentage change (APC) was significantly different from zero at α = 0.05.

Nonmetropolitan areas consistently had higher AAMRs than metropolitan areas (8.9; 95% CI, 8.7-9.1; vs 7.5; 95% CI, 7.4-7.6 per 1,000,000). Nonmetropolitan areas showed a nonsignificant AAMR increase from 1999 to 2018 (*P* = .058), followed by a significant increase from 2018 to 2020 (*P* < .001). Metropolitan areas had a significant increase in AAMR from 1999 to 2018 (*P* = .009), followed by a sharper rise from 2018 to 2020 (*P* < .001). The details are depicted in Figure 7 and Supplementary Tables 2 and 8.

### 3.8 Trends comparison

#### 3.8.1 PE alone vs combined mortality

**Figure 8:**
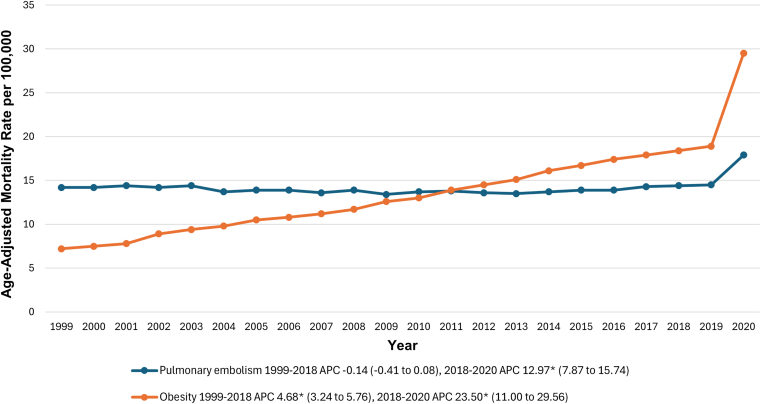
Individual pulmonary embolism and obesity-related age-adjusted mortality rates per 100,000 in adults in the United States, 1999-2020. ∗The annual percentage change (APC) was significantly different from zero at α = 0.05.

The AAMR due to PE was 14.2 (95% CI, 14.0-14.3) per 100,000 individuals in 1999, increasing to 17.9 (95% CI, 17.7-18.1) in 2020. From 1999 to 2018, AAMRs for PE remained stable (APC, −0.14; 95% CI, −0.41 to 0.08; *P* = .206). However, from 2018 to 2020, a sharp increase was observed (APC, 12.97; 95% CI, 7.87-15.74; *P* < .001) (Figure 8 and Supplementary Tables 2 and 9). Throughout the study period (1999-2020), the increase in AAMR was significantly greater for combined PE and obesity compared with PE alone (AAPCD, 3.61; 95% CI, 2.91-4.32; p < 0.001) (Supplementary Table 10).

#### 3.8.2 Obesity alone vs combined mortality

The AAMR for obesity was 7.2 (95% CI, 7.1-7.3) in 1999, increasing to 29.5 (95% CI, 29.3-29.7) per 100,000 in 2020. From 1999 to 2018, AAMRs showed a significant increase (APC, 4.68; 95% CI, 3.24-5.76; *P* = .005), followed by another significant sharp rise from 2018 to 2020 (APC, 23.50; 95% CI, 11.00 to 29.56; *P* < .001) (Figure 8 and Supplementary Tables 2 and 9). From 1999 to 2020, the increase in mortality from combined PE and obesity was significantly lower than the increase in mortality from obesity alone (AAPCD: −2.14; 95% CI, −3.27 to −1.02; *P* < .001) (Supplementary Table 10).

## 4 Discussion

Our study has several key findings that highlight trends in mortality among individuals with PE and concurrent obesity. First, between 1999 and 2020, we observed a significant upward trend in AAMR, with a steady increase from 1999 to 2018, followed by a sharper increase from 2018 to 2020. Second, women consistently had higher AAMRs than men. Third, middle-aged adults had higher rates than younger and older adults. Fourth, NH Black individuals exhibited higher AAMRs than other ethnoracial groups. Finally, residents of nonmetropolitan areas had higher AAMRs than those in metropolitan areas.

Prior studies have examined temporal trends in PE-related mortality in the United States. Ogunsola et al. [6] reported that the overall age-adjusted mortality rate from PE decreased from 3.08 to 2.30 per 100,000 between 1999 and 2020, with an annual decline from 1999 to 2009 followed by a plateau thereafter. In contrast, our results for PE with obesity demonstrated a steady increase in mortality from 1999 to 2018, followed by an even sharper increase from 2018 to 2020, suggesting that the obesity epidemic has shifted the trajectory of PE-related deaths in this subgroup. Cash et al. [19] further analyzed crude PE-related mortality rates across age groups, showing significant increases among individuals aged 25 to 69 years, whereas declines were observed in those aged ≥70 years. In comparison, our age-stratified analysis revealed that mortality from PE with obesity was the highest in middle-aged adults, followed by older adults and young adults, with all groups demonstrating a consistent upward trend that accelerated after 2018. These contrasts with overall declining or plateauing PE-related mortality highlight how age-specific patterns differ in the PE with obesity subgroup.

Our results demonstrate that the rise in mortality from combined PE and obesity closely parallels the upward trajectory of obesity-related mortality, suggesting that the obesity burden is a major driver of this trend. These findings, derived from a nationwide analysis, contrast with prior studies reporting a paradoxical association between obesity and lower PE-related mortality despite obesity’s well-established role as a risk factor for PE [20][]. Bauer [21] further observed that the reduction in PE-related mortality among obese patients was limited to those aged ≥40 years and may reflect confounders such as lower smoking prevalence or greater metabolic reserve rather than a true protective effect. Taken together, while prior studies suggest context-dependent protective associations of obesity at the individual level, our population-level analysis indicates that the increasing prevalence of obesity substantially contributes to the increased mortality observed in patients with concurrent PE and obesity. Furthermore, we hypothesize that the overall increased risk of PE in obese patients [1,3] likely outweighs any small protective effect of obesity on PE-related mortality, with the higher prevalence of obesity ultimately leading to increased mortality from PE with obesity when examining decade-long trends, as in our study. Another noteworthy finding from our analysis was that the magnitude of increase in mortality among individuals with concurrent PE and obesity was lower than that observed for obesity alone. This finding may be plausibly explained by the fact that obesity-related mortality is driven by multiple comorbid conditions beyond PE, including coronary artery disease, heart failure, and sudden cardiac death [22]. The strong association of obesity with these conditions likely contributes to a higher overall mortality rate among patients with obesity alone compared with those with both PE and obesity. Additionally, potential factors such as misclassification or inaccurate coding on death certificates, as well as inherent limitations of the database, may have influenced these findings.

Our study reported that females had significantly higher AAMR than males, consistent with the findings of several studies showing higher short-term PE-related mortality in females [23,24]. However, in studies controlling for disease severity, this sex difference was not significant, and other studies have found that PE recurrence is more common in males than that in females and that the latter had better long-term survival rates [23,25]. It is believed that females are at higher risk of mortality from PE due to factors such as increased subcutaneous fat and comorbid conditions like systemic lupus erythematosus and rheumatoid arthritis—both of which are risk factors for PE and are more common in females—in addition to health care disparities [26].

Our results demonstrate the highest mortality rate among NH Black individuals. Ethnoracial and geographical trends are primarily driven by differences in socioeconomic status, where comorbidities that put patients at higher risk for mortality appear to be better managed in urban areas compared with rural or nonmetropolitan regions, as well as among certain racial groups [25]. However, as demonstrated by Sathianathan et al. [27], Black and Hispanic patients tend to present with PE at a younger age than White patients, suggesting that differences in mortality may also be due to earlier disease progression in some racial groups, underscoring the need for equitable management [28]. As noted in the review by Huisman et al. [1], differences in the prevalence of certain risk factors, such as sickle cell trait, may contribute to the higher risk of PE among Black populations. Supporting this, a large US-based prospective cohort study found that African American individuals with sickle cell trait had approximately a 2-fold increased risk of PE compared with those without the trait [29]. Similarly, a study by Phillips et al. [28] on the severity of PE in Black patients found that Black race was associated with higher severity of PE and younger presentation compared with White patients. However, they did not find a significant difference in in-hospital mortality risk between the 2 groups [28].

### 4.1 Clinical perspectives

Our study highlights the increasing burden of PE- and obesity-related mortality, particularly among middle-aged adults, women, and minority groups. Physicians should prioritize identifying and addressing modifiable risk factors that contribute to both obesity and PE. Early intervention, especially in young adults, could prevent disease progression and reduce long-term mortality risk. The sex-specific, racial, and urbanization status disparities observed in our study highlight the need for equitable healthcare strategies and prompt public health action. A tailored, multidisciplinary approach that combines preventive care, equitable access, and innovative treatments will be essential for optimizing outcomes and reducing PE- and obesity-related mortality. Although evidence on the impact of antiobesity medications on index and recurrent thromboembolic events remains limited, it is generally hypothesized that weight reduction achieved through these agents may mitigate systemic inflammation and prothrombotic activity [30]. Future studies should investigate the potential role of these therapies in patients with PE and concomitant obesity and evaluate their effects on mortality and long-term outcomes. Understanding how antiobesity medications affect patient outcomes could help clinicians refine treatment protocols.

### 4.2 Limitations

Our study has several limitations. First, the use of the multiple cause of death feature in the CDC WONDER database relies on the Boolean operator “AND,” which excludes cases where only PE or obesity is mentioned as a cause of death. This accounts for the relatively low total of 35,211 deaths. However, this limitation is inherent to the database itself, and analyzing trends in patients with coexisting diseases remains important from a public health perspective. Second, relying solely on mortality data may overlook the significant morbidity associated with PE and obesity, as nonfatal cases can profoundly impact quality of life and health care utilization. Third, the ICD-10 codes in the CDC WONDER datasets focus on diagnostic information rather than detailed clinical data or patient characteristics. While these codes provide a general overview of a patient’s condition, they may omit important factors such as comorbidities, disease severity, treatment protocols, socioeconomic status, and access to health care—all of which can influence mortality. Furthermore, ICD-10 coding used in the CDC WONDER database does not differentiate between obesity severity or stages (eg, class I-III), limiting our ability to explore dose-response relationships between obesity- and PE-related mortality. Fourth, the dataset lacks information on a cardiovascular or thromboembolic history, limiting our understanding of underlying conditions that contribute to PE. Moreover, it does not distinguish between first-time and recurrent PE events, limiting the ability to assess whether mortality was related to inadequate primary prevention or suboptimal anticoagulant therapy. Fifth, the absence of data on medical therapy further restricts insights into treatment effectiveness and its impact on mortality, which is crucial for improving outcomes. Sixth, the database does not have features to adjust for or match baseline comorbidities and risk factors for PE. Seventh, while our analysis is adjusted for age, we acknowledge that changes in the sex and race composition of the US population over time, as well as potential interactions between age, sex, and race, may have potentially contributed to the observed mortality patterns. Finally, the dataset’s focus on the US population limits the generalizability of our findings to other regions. Future research should aim to incorporate international data to increase the applicability of the results to diverse populations while considering clinical and socioeconomic factors.

## 5 Conclusion

The significant rise in mortality related to PE and obesity from 1999 to 2020, with a sharper increase since 2018, highlights major public health concerns. Women, middle-aged adults, NH Black individuals, and those in nonmetropolitan areas experienced higher AAMRs, underscoring the need for targeted interventions to address these disparities. Future efforts should prioritize improving health care access in nonmetropolitan areas. Additionally, further research into the social and health care factors driving these trends is essential for developing effective and equitable prevention and management strategies.
