# Supplementary material for: Disparities and trends in pulmonary embolism mortality with and without obesity: a nationwide US analysis
Source: Res Pract Thromb Haemost. 2025 Oct 30;9(8):103240. doi: 10.1016/j.rpth.2025.103240 (PMC12702333; doi:10.1016/j.rpth.2025.103240)
Supplement: Supplementary File [file mmc1.docx]

**Supplemental Table 1** Overall Pulmonary Embolism and Obesity -related Mortality per 1,000,000 Adults in the United States, 1999 to 2020.

| **Year** | **Deaths** | **Age-Adjusted Mortality Rate (95% CI)** | **Population** |
| --- | --- | --- | --- |
| 1999 | 904 | 5.1 (4.7-5.4) | 180408769 |
| 2000 | 921 | 5.1 (4.8-5.4) | 181984640 |
| 2001 | 980 | 5.3 (5.0-5.7) | 184305128 |
| 2002 | 1085 | 5.8 (5.4-6.1) | 186208028 |
| 2003 | 1174 | 6.2 (5.8-6.6) | 188090429 |
| 2004 | 1118 | 5.8 (5.5-6.1) | 190205384 |
| 2005 | 1169 | 6.0 (5.6-6.3) | 192551384 |
| 2006 | 1230 | 6.2 (5.8-6.5) | 195019359 |
| 2007 | 1274 | 6.3 (5.9-6.6) | 197403777 |
| 2008 | 1365 | 6.7 (6.3-7.0) | 199795090 |
| 2009 | 1433 | 6.9 (6.6-7.3) | 202107016 |
| 2010 | 1610 | 7.7 (7.3-8.1) | 203891983 |
| 2011 | 1664 | 7.9 (7.5-8.3) | 206592936 |
| 2012 | 1702 | 8.0 (7.6-8.4) | 208826037 |
| 2013 | 1713 | 7.9 (7.5-8.3) | 211085314 |
| 2014 | 1976 | 9.0 (8.6-9.4) | 213809280 |
| 2015 | 1994 | 9.0 (8.6-9.4) | 216553817 |
| 2016 | 2054 | 9.2 (8.8-9.6) | 218641417 |
| 2017 | 2107 | 9.3 (8.9-9.7) | 221447331 |
| 2018 | 2171 | 9.5 (9.1-9.9) | 223311190 |
| 2019 | 2329 | 10.1 (9.7-10.5) | 224981167 |
| 2020 | 3238 | 13.9 (13.4-14.4) | 226635013 |
| Total | 35211 | 7.7 (7.6-7.8) | 4,473,854,489 |

**Supplemental Table 2** Annual percent change (APC) of Pulmonary Embolism and Obesity –related Age-Adjusted Mortality Rates per 1,000,000 in Adults in the United States, 1999 to 2020

| **Year Interval** | **APC (95% CI)** | **p-value** |
| --- | --- | --- |
| **Overall** | | |
| 1999-2018 | 3.45 (2.65 to 4.01) | 0.005 |
| 2018-2020 | 16.28 (6.24 to 21.22) | <0.001 |
| **Men** | | |
| 1999-2018 | 4.86 (3.57 to 5.61) | 0.007 |
| 2018-2020 | 18.78 (6.59 to 25.27) | <0.001 |
| **Women** | | |
| 1999-2018 | 2.61 (1.47 to 3.25) | 0.015 |
| 2018-2020 | 15.48 (4.38 to 20.97) | <0.001 |
| **Young Adults (25-44 years)** | | |
| 1999-2018 | 3.84 (0.50 to 11.26) | 0.042 |
| 2018-2020 | 16.12 (4.06 to 23.48) | 0.001 |
| **Middle Aged Adults (45-64 years)** | | |
| 1999-2018 | 3.14 (2.45 to 3.70) | 0.002 |
| 2018-2020 | 20.75 (9.66 to 26.36) | <0.001 |
| **Older Adults (65 years and above)** | | |
| 1999-2018 | 3.51 (2.04 to 4.18) | 0.016 |
| 2018-2020 | 14.02 (4.85 to 19.21) | <0.001 |
| **NH Black or African American** | | |
| 1999-2018 | 2.95 (2.22 to 3.56) | 0.002 |
| 2018-2020 | 20.88 (10.20 to 27.49) | <0.001 |
| **Hispanic or Latino** | | |
| 1999-2018 | 2.49 (-0.16 to 4.14) | 0.057 |
| 2018-2020 | 27.81 (6.22 to 38.86) | <0.001 |
| **NH White** | | |
| 1999-2018 | 4.01 (2.78 to 4.65) | 0.009 |
| 2018-2020 | 14.90 (5.05 to 19.34) | <0.001 |
| **NH American Indian or Alaska Native** | | |
| 1999-2020 | NA | NA |
| **NH Asian or Pacific Islander** | | |
| 1999-2020 | NA | NA |
| **Metropolitan area** | | |
| 1999-2018 | 3.38 (2.34 to 4.04) | 0.009 |
| 2018-2020 | 16.52 (5.40 to 21.85) | <0.001 |
| **Nonmetropolitan area** | | |
| 1999-2018 | 4.14 (-0.47 to 9.66) | 0.058 |
| 2018-2020 | 16.47 (4.53 to 24.19) | <0.001 |
| **Northeast** | | |
| 1999-2018 | 3.73 (-1.55 to 13.79) | 0.078 |
| 2018-2020 | 13.31 (3.77 to 20.15) | 0.003 |
| **Midwest** | | |
| 1999-2018 | 4.22 (-0.20 to 9.69) | 0.054 |
| 2018-2020 | 15.36 (4.51 to 22.50) | <0.001 |
| **South** | | |
| 1999-2018 | 3.21 (2.63 to 3.78) | <0.001 |
| 2018-2020 | 20.71 (11.20 to 25.34) | <0.001 |
| **West** | | |
| 1999-2018 | 2.99 (2.03 to 3.72) | 0.0064 |
| 2018-2020 | 18.15 (6.65 to 24.26) | <0.001 |
| **Pulmonary Embolism** | | |
| 1999-2018 | -0.14 (-0.41 to 0.08) | 0.206 |
| 2018-2020 | 12.97 (7.87 to 15.74) | <0.001 |
| **Obesity** | | |
| 1999-2018 | 4.68 (3.24 to 5.76) | 0.005 |
| 2018-2020 | 23.50 (11.00 to 29.56) | <0.001 |

APC = Annual percent change; NH = non-Hispanic. N/A = unreliable or suppressed

**Supplemental Table 3** Pulmonary Embolism and Obesity -related Mortality, Stratified by Sex per 1,000,000 Adults in the United States, 1999 to 2020.

| **Year** | **Deaths** | | **Age-adjusted Mortality Rate (95 % CI)** | |
| --- | --- | --- | --- | --- |
|  | **Women** | **Men** | **Men** | **Women** |
| 1999 | 585 | 319 | 3.7 (3.3-4.1) | 6.2 (5.7-6.7) |
| 2000 | 584 | 337 | 3.9 (3.5-4.3) | 6.2 (5.7-6.7) |
| 2001 | 663 | 317 | 3.6 (3.2-4.0) | 6.8 (6.3-7.4) |
| 2002 | 720 | 365 | 4.0 (3.6-4.5) | 7.4 (6.8-7.9) |
| 2003 | 782 | 392 | 4.3 (3.9-4.7) | 7.9 (7.3-8.4) |
| 2004 | 675 | 443 | 4.7 (4.3-5.2) | 6.7 (6.2-7.2) |
| 2005 | 733 | 436 | 4.6 (4.2-5.1) | 7.2 (6.6-7.7) |
| 2006 | 770 | 460 | 4.8 (4.3-5.2) | 7.4 (6.9-7.9) |
| 2007 | 810 | 464 | 4.7 (4.3-5.2) | 7.7 (7.1-8.2) |
| 2008 | 782 | 583 | 5.9 (5.4-6.4) | 7.3 (6.8-7.9) |
| 2009 | 879 | 554 | 5.5 (5.0-6.0) | 8.2 (7.6-8.7) |
| 2010 | 940 | 670 | 6.7 (6.2-7.2) | 8.6 (8.0-9.1) |
| 2011 | 976 | 688 | 6.7 (6.2-7.2) | 9.0 (8.4-9.5) |
| 2012 | 1038 | 664 | 6.4 (5.9-6.9) | 9.4 (8.9-10.0) |
| 2013 | 989 | 724 | 6.9 (6.4-7.4) | 8.8 (8.2-9.4) |
| 2014 | 1142 | 834 | 7.8 (7.3-8.4) | 10.1 (9.5-10.7) |
| 2015 | 1111 | 883 | 8.1 (7.6-8.7) | 9.8 (9.2-10.4) |
| 2016 | 1171 | 883 | 8.1 (7.6-8.7) | 10.2 (9.6-10.8) |
| 2017 | 1189 | 918 | 8.3 (7.7-8.8) | 10.2 (9.6-10.8) |
| 2018 | 1203 | 968 | 8.7 (8.2-9.3) | 10.2 (9.6-10.8) |
| 2019 | 1316 | 1013 | 9.1 (8.5-9.6) | 11.0 (10.4-11.7) |
| 2020 | 1743 | 1495 | 13.2 (12.6-13.9) | 14.6 (13.8-15.3) |
| **Total** | 20801 | 14410 | 6.5 (6.4-6.6) | 8.8 (8.6-8.9) |

**Supplemental Table 4** Pulmonary Embolism and Obesity -related Mortality per 1,000,000, Stratified by Age group in Adults in the United States, 1999 to 2020.

|  | **Deaths** | | | **Age-Adjusted Rate (95% CI)** | | |
| --- | --- | --- | --- | --- | --- | --- |
| **Year** | **Young Adults** | **Middle Aged Adults** | **Older Adults** | **Young Adults** | **Middle Aged Adults** | **Older Adults** |
| 1999 | 294 | 408 | 202 | 3.5 (3.1-3.9) | 6.8 (6.1-7.4) | 5.8 (5.0-6.6) |
| 2000 | 325 | 392 | 204 | 3.9 (3.4-4.3) | 6.3 (5.7-6.9) | 5.8 (5.0-6.6) |
| 2001 | 289 | 474 | 217 | 3.4 (3.0-3.8) | 7.4 (6.7-8.0) | 6.2 (5.3-7.0) |
| 2002 | 345 | 525 | 215 | 4.1 (3.7-4.6) | 7.8 (7.2-8.5) | 6.1 (5.3-6.9) |
| 2003 | 374 | 541 | 259 | 4.5 (4.1-5.0) | 7.8 (7.2-8.5) | 7.2 (6.4-8.1) |
| 2004 | 318 | 563 | 237 | 3.9 (3.5-4.3) | 7.9 (7.3-8.6) | 6.6 (5.7-7.4) |
| 2005 | 353 | 560 | 256 | 4.3 (3.8-4.7) | 7.6 (7.0-8.3) | 7.0 (6.2-7.9) |
| 2006 | 352 | 619 | 259 | 4.3 (3.9-4.8) | 8.1 (7.5-8.8) | 7.0 (6.1-7.9) |
| 2007 | 377 | 663 | 234 | 4.6 (4.2-5.1) | 8.6 (7.9-9.2) | 6.2 (5.4-7.0) |
| 2008 | 365 | 664 | 336 | 4.5 (4.1-5.0) | 8.4 (7.8-9.0) | 8.6 (7.7-9.6) |
| 2009 | 408 | 708 | 317 | 5.1 (4.6-5.6) | 8.8 (8.1-9.4) | 8.0 (7.1-8.9) |
| 2010 | 420 | 825 | 365 | 5.3 (4.8-5.8) | 10.0 (9.3-10.7) | 9.1 (8.2-10.0) |
| 2011 | 472 | 804 | 388 | 5.9 (5.4-6.5) | 9.6 (8.9-10.3) | 9.4 (8.5-10.3) |
| 2012 | 496 | 807 | 399 | 6.3 (5.7-6.8) | 9.6 (8.9-10.3) | 9.2 (8.3-10.1) |
| 2013 | 460 | 849 | 404 | 5.7 (5.2-6.2) | 10.2 (9.5-10.9) | 9.1 (8.2-10.0) |
| 2014 | 505 | 989 | 482 | 6.3 (5.8-6.9) | 11.8 (11.0-12.5) | 10.5 (9.5-11.4) |
| 2015 | 542 | 971 | 481 | 6.7 (6.1-7.3) | 11.5 (10.7-12.2) | 9.9 (9.0-10.8) |
| 2016 | 567 | 971 | 516 | 7.0 (6.4-7.6) | 11.6 (10.8-12.3) | 10.3 (9.4-11.2) |
| 2017 | 584 | 998 | 525 | 7.0 (6.5-7.6) | 11.8 (11.0-12.5) | 10.2 (9.3-11.1) |
| 2018 | 580 | 991 | 600 | 7.0 (6.4-7.6) | 11.8 (11.0-12.5) | 11.3 (10.4-12.2) |
| 2019 | 603 | 1096 | 630 | 7.2 (6.6-7.8) | 13.1 (12.3-13.9) | 11.5 (10.6-12.4) |
| 2020 | 875 | 1523 | 840 | 10.3 (9.7-11.0) | 18.3 (17.3-19.2) | 14.8 (13.8-15.8) |
| **Overall** | 9904 | 16941 | 8366 | 5.5 (5.3-5.6) | 10.0 (9.8-10.1) | 9.0 (8.8-9.1) |

Young Adult = 25-44 years; Middle Aged Adults = 45-64 years; Older Adults = 65 years and above; N/A = unreliable or suppressed

**Supplemental Table 5** Pulmonary Embolism and Obesity -related Age-Adjusted Mortality Rates per 1,000,000, Stratified by Race in Adults in the United States from 1999 to 2020

| **Deaths** | | | | | | **Age-Adjusted Rate (95% CI)** | | | | |
| --- | --- | --- | --- | --- | --- | --- | --- | --- | --- | --- |
| **Year** | **NH White** | **NH Black or African American** | **NH American Indian or Alaska Native** | **Hispanic or Latino** | **NH Asian or Pacific Islander** | **NH White** | **NH Black or African American** | **NH American Indian or Alaska Native** | **Hispanic or Latino** | **NH Asian or Pacific Islander** |
| 1999 | 640 | 217 | N/A | 36 | N/A | 4.7 (4.3-5.0) | 11.4 (9.8-12.9) | N/A (N/A-N/A) | 2.3 (1.6-3.3) | N/A (N/A-N/A) |
| 2000 | 630 | 228 | N/A | 54 | N/A | 4.6 (4.2-4.9) | 11.8 (10.2-13.3) | N/A (N/A-N/A) | 3.6 (2.6-4.8) | N/A (N/A-N/A) |
| 2001 | 676 | 240 | N/A | 54 | N/A | 4.8 (4.5-5.2) | 12.0 (10.5-13.5) | N/A (N/A-N/A) | 3.5 (2.6-4.7) | N/A (N/A-N/A) |
| 2002 | 736 | 277 | N/A | 60 | N/A | 5.2 (4.8-5.6) | 13.4 (11.8-15.0) | N/A (N/A-N/A) | 3.5 (2.6-4.5) | N/A (N/A-N/A) |
| 2003 | 825 | 286 | N/A | 50 | N/A | 5.8 (5.4-6.2) | 13.7 (12.1-15.3) | N/A (N/A-N/A) | 3.1 (2.2-4.1) | N/A (N/A-N/A) |
| 2004 | 762 | 277 | N/A | 63 | N/A | 5.3 (4.9-5.7) | 13.2 (11.7-14.8) | N/A (N/A-N/A) | 3.5 (2.7-4.6) | N/A (N/A-N/A) |
| 2005 | 799 | 291 | 11 | 61 | N/A | 5.5 (5.1-5.9) | 13.6 (12.0-15.1) | N/A (4.0-14.4) | 3.1 (2.3-4.0) | N/A (N/A-N/A) |
| 2006 | 833 | 319 | N/A | 67 | N/A | 5.6 (5.3-6.0) | 14.3 (12.7-15.9) | N/A (N/A-N/A) | 3.2 (2.5-4.2) | N/A (N/A-N/A) |
| 2007 | 866 | 313 | N/A | 80 | N/A | 5.9 (5.5-6.3) | 14.0 (12.4-15.6) | N/A (N/A-N/A) | 3.9 (3.1-4.9) | N/A (N/A-N/A) |
| 2008 | 941 | 330 | N/A | 82 | N/A | 6.4 (6.0-6.8) | 14.5 (12.9-16.1) | N/A (N/A-N/A) | 3.8 (2.9-4.8) | N/A (N/A-N/A) |
| 2009 | 988 | 331 | N/A | 84 | 11 | 6.7 (6.3-7.1) | 14.4 (12.9-16.0) | N/A (N/A-N/A) | 3.8 (3.0-4.7) | N/A (0.5-1.9) |
| 2010 | 1089 | 391 | 12 | 108 | N/A | 7.3 (6.8-7.7) | 16.6 (14.9-18.2) | N/A (4.0-13.7) | 4.6 (3.6-5.5) | N/A (N/A-N/A) |
| 2011 | 1120 | 403 | 10 | 117 | N/A | 7.5 (7.0-7.9) | 17.0 (15.3-18.7) | N/A (2.7-10.5) | 4.6 (3.7-5.4) | N/A (N/A-N/A) |
| 2012 | 1160 | 405 | 13 | 106 | 11 | 7.8 (7.3-8.3) | 16.7 (15.0-18.4) | N/A (4.7-15.1) | 4.1 (3.2-4.9) | N/A (0.5-2) |
| 2013 | 1150 | 431 | N/A | 107 | N/A | 7.6 (7.1-8.0) | 17.5 (15.9-19.2) | N/A (N/A-N/A) | 3.9 (3.1-4.7) | N/A (N/A-N/A) |
| 2014 | 1288 | 510 | 13 | 139 | 17 | 8.4 (7.9-8.9) | 20.5 (18.7-22.3) | N/A (4.0-13.5) | 5.0 (4.1-5.8) | N/A (0.8-2.2) |
| 2015 | 1345 | 473 | N/A | 140 | 16 | 8.7 (8.2-9.2) | 18.6 (16.9-20.3) | N/A (N/A-N/A) | 4.7 (3.9-5.5) | N/A (0.7-2.1) |
| 2016 | 1389 | 493 | 10 | 139 | 12 | 9.2 (8.7-9.7) | 19.1 (17.4-20.8) | N/A (3.0-11.6) | 4.6 (3.8-5.4) | N/A (0.5-1.5) |
| 2017 | 1381 | 532 | 16 | 154 | 15 | 9.0 (8.5-9.5) | 20.1 (18.4-21.9) | N/A (4.8-14.2) | 4.7 (4.0-5.5) | N/A (0.6-1.7) |
| 2018 | 1460 | 518 | 16 | 161 | 14 | 9.5 (9.0-10.0) | 19.5 (17.8-21.3) | N/A (5.5-16.1) | 5.1 (4.3-5.9) | N/A (0.5-1.6) |
| 2019 | 1558 | 588 | 12 | 147 | 15 | 10.1 (9.5-10.6) | 21.6 (19.8-23.4) | N/A (3.4-12.3) | 4.4 (3.7-5.2) | N/A (0.6-1.7) |
| 2020 | 2028 | 854 | 19 | 301 | 28 | 13.0 (12.4-13.6) | 30.9 (28.8-33.0) | N/A (6.7-17.9) | 8.6 (7.6-9.6) | 1.9 (1.3-2.7) |
| **Overall** | 23664 | 8707 | 194 | 2310 | 206 | 7.2 (7.1-7.3) | 16.8 (16.5-17.2) | 5.9 (5.0-6.7) | 4.4 (4.2-4.6) | 0.9 (0.8-1) |

NH=non-Hispanic; N/A = unreliable or suppressed

**Supplemental Table 6** Pulmonary Embolism and Obesity -related Age-Adjusted Mortality Rates per 1,000,000, Stratified by State in Adults in the United States, 1999 to 2020.

| **State** | **Age-Adjusted Rate (95% CI)** |
| --- | --- |
| Alabama | 4.6 (4.1-5.1) |
| Alaska | 5.1 (3.6-6.8) |
| Arizona | 6.9 (6.4-7.4) |
| Arkansas | 7.4 (6.5-8.2) |
| California | 7.6 (7.3-7.8) |
| Colorado | 9.8 (9-10.5) |
| Connecticut | 4.8 (4.2-5.4) |
| Delaware | 10.7 (8.9-12.5) |
| District of Columbia | 19.8 (16.8-22.7) |
| Florida | 6.2 (5.9-6.5) |
| Georgia | 5.3 (4.9-5.7) |
| Hawaii | 5.3 (4.2-6.3) |
| Idaho | 7.5 (6.4-8.6) |
| Illinois | 7.1 (6.8-7.5) |
| Indiana | 7.9 (7.3-8.5) |
| Iowa | 9.5 (8.5-10.4) |
| Kansas | 10.1 (9.1-11) |
| Kentucky | 7.3 (6.6-8) |
| Louisiana | 8.8 (8-9.5) |
| Maine | 4.2 (3.4-5.2) |
| Maryland | 7.7 (7.1-8.3) |
| Massachusetts | 4.3 (3.9-4.7) |
| Michigan | 9.1 (8.6-9.5) |
| Minnesota | 9.4 (8.7-10.1) |
| Mississippi | 9.8 (8.9-10.8) |
| Missouri | 6.8 (6.2-7.3) |
| Montana | 7.7 (6.3-9.1) |
| Nebraska | 6.7 (5.7-7.7) |
| Nevada | 5.4 (4.7-6.2) |
| New Hampshire | 6.5 (5.4-7.6) |
| New Jersey | 6.3 (5.9-6.8) |
| New Mexico | 7.9 (6.9-9) |
| New York | 9.4 (9.1-9.8) |
| North Carolina | 8 (7.6-8.5) |
| North Dakota | 10.5 (8.4-12.6) |
| Ohio | 9 (8.6-9.5) |
| Oklahoma | 11.4 (10.5-12.3) |
| Oregon | 5.7 (5.1-6.3) |
| Pennsylvania | 6 (5.7-6.4) |
| Rhode Island | 6.8 (5.5-8.1) |
| South Carolina | 8 (7.3-8.6) |
| South Dakota | 7.7 (6.2-9.5) |
| Tennessee | 8.6 (8-9.2) |
| Texas | 9.6 (9.3-9.9) |
| Utah | 10.2 (9.1-11.3) |
| Vermont | 13 (10.7-15.3) |
| Virginia | 4.3 (4-4.7) |
| Washington | 7.9 (7.3-8.4) |
| West Virginia | 8.6 (7.5-9.8) |
| Wisconsin | 11 (10.3-11.7) |
| Wyoming | 13 (10.5-15.5) |
| **Overall** | 7.7 (7.6-7.8) |

**Supplemental Table 7** Pulmonary Embolism and Obesity -related Age-Adjusted Mortality Rate per 1,000,000, Stratified by Census Region in Adults in the United States 1999-2020.

| **Census Region** | **Year** | **Age-Adjusted Rate (95% CI)** |
| --- | --- | --- |
| Northeast | 1999 | 4.8 (4.1-5.5) |
| Northeast | 2000 | 4.5 (3.8-5.2) |
| Northeast | 2001 | 5.0 (4.2-5.7) |
| Northeast | 2002 | 5.4 (4.7-6.2) |
| Northeast | 2003 | 5.1 (4.4-5.9) |
| Northeast | 2004 | 4.9 (4.2-5.7) |
| Northeast | 2005 | 5.2 (4.4-5.9) |
| Northeast | 2006 | 5.4 (4.6-6.1) |
| Northeast | 2007 | 5.8 (5.0-6.5) |
| Northeast | 2008 | 6.5 (5.7-7.3) |
| Northeast | 2009 | 6.4 (5.6-7.3) |
| Northeast | 2010 | 7.6 (6.7-8.4) |
| Northeast | 2011 | 8.0 (7.0-8.9) |
| Northeast | 2012 | 7.5 (6.7-8.4) |
| Northeast | 2013 | 7.5 (6.7-8.4) |
| Northeast | 2014 | 8.9 (7.9-9.8) |
| Northeast | 2015 | 8.2 (7.3-9.1) |
| Northeast | 2016 | 8.7 (7.8-9.7) |
| Northeast | 2017 | 8.5 (7.6-9.4) |
| Northeast | 2018 | 8.4 (7.5-9.3) |
| Northeast | 2019 | 9.5 (8.5-10.5) |
| Northeast | 2020 | 12.2 (11.1-13.3) |
| Northeast | **Overall** | 7.0 (6.8-7.2) |
| Midwest | 1999 | 5.8 (5.0-6.5) |
| Midwest | 2000 | 5.0 (4.3-5.6) |
| Midwest | 2001 | 4.7 (4.0-5.4) |
| Midwest | 2002 | 6.1 (5.4-6.9) |
| Midwest | 2003 | 7.0 (6.2-7.7) |
| Midwest | 2004 | 6.3 (5.6-7.1) |
| Midwest | 2005 | 7.0 (6.2-7.7) |
| Midwest | 2006 | 6.7 (5.9-7.5) |
| Midwest | 2007 | 6.9 (6.1-7.7) |
| Midwest | 2008 | 7.5 (6.6-8.3) |
| Midwest | 2009 | 8.2 (7.3-9.0) |
| Midwest | 2010 | 8.7 (7.8-9.6) |
| Midwest | 2011 | 8.5 (7.7-9.4) |
| Midwest | 2012 | 8.5 (7.6-9.3) |
| Midwest | 2013 | 9.0 (8.1-9.9) |
| Midwest | 2014 | 10.4 (9.5-11.4) |
| Midwest | 2015 | 10.7 (9.8-11.7) |
| Midwest | 2016 | 10.8 (9.8-11.7) |
| Midwest | 2017 | 10.8 (9.9-11.8) |
| Midwest | 2018 | 11.7 (10.7-12.8) |
| Midwest | 2019 | 10.6 (9.6-11.5) |
| Midwest | 2020 | 16.3 (15.1-17.5) |
| Midwest | **Overall** | 8.6 (8.4-8.8) |
| South | 1999 | 4.8 (4.3-5.3) |
| South | 2000 | 5.4 (4.8-5.9) |
| South | 2001 | 5.9 (5.3-6.5) |
| South | 2002 | 5.8 (5.3-6.4) |
| South | 2003 | 6.2 (5.7-6.8) |
| South | 2004 | 6.1 (5.5-6.7) |
| South | 2005 | 5.8 (5.2-6.3) |
| South | 2006 | 6.3 (5.7-6.9) |
| South | 2007 | 6.1 (5.5-6.7) |
| South | 2008 | 6.3 (5.8-6.9) |
| South | 2009 | 6.6 (6.1-7.2) |
| South | 2010 | 7.1 (6.5-7.7) |
| South | 2011 | 7.2 (6.6-7.8) |
| South | 2012 | 7.9 (7.3-8.6) |
| South | 2013 | 7.7 (7.0-8.3) |
| South | 2014 | 8.6 (8.0-9.3) |
| South | 2015 | 8.6 (7.9-9.2) |
| South | 2016 | 9.1 (8.4-9.8) |
| South | 2017 | 9.0 (8.4-9.7) |
| South | 2018 | 9.3 (8.6-10.0) |
| South | 2019 | 10.5 (9.8-11.2) |
| South | 2020 | 13.8 (13.0-14.6) |
| South | **Overall** | 7.6 (7.5-7.7) |
| West | 1999 | 5.0 (4.2-5.7) |
| West | 2000 | 5.3 (4.6-6.1) |
| West | 2001 | 5.3 (4.6-6.0) |
| West | 2002 | 5.7 (5.0-6.4) |
| West | 2003 | 6.3 (5.6-7.1) |
| West | 2004 | 5.5 (4.8-6.2) |
| West | 2005 | 6.0 (5.3-6.7) |
| West | 2006 | 6.2 (5.4-6.9) |
| West | 2007 | 6.4 (5.7-7.2) |
| West | 2008 | 6.6 (5.9-7.4) |
| West | 2009 | 6.7 (5.9-7.4) |
| West | 2010 | 7.6 (6.9-8.4) |
| West | 2011 | 8.3 (7.5-9.2) |
| West | 2012 | 8.1 (7.3-8.9) |
| West | 2013 | 7.6 (6.8-8.4) |
| West | 2014 | 8.4 (7.6-9.3) |
| West | 2015 | 8.6 (7.8-9.4) |
| West | 2016 | 8.5 (7.7-9.3) |
| West | 2017 | 9.1 (8.2-9.9) |
| West | 2018 | 8.6 (7.8-9.4) |
| West | 2019 | 9.4 (8.6-10.3) |
| West | 2020 | 13.4 (12.4-14.3) |
| West | **Overall** | 7.6 (7.4-7.7) |

**Supplemental Table 8** Pulmonary Embolism and Obesity –related Age-Adjusted Mortality Rates per 1,000,000, Stratified by Urbanization Status in Adults in the United States, 1999 to 2020

| **Year** | **Metropolitan** | **Rural/non-metropolitan** |
| --- | --- | --- |
| 1999 | 4.9 (4.5-5.3) | 5.8 (4.9-6.7) |
| 2000 | 4.9 (4.6-5.3) | 6.0 (5.1-6.9) |
| 2001 | 5.2 (4.9-5.6) | 5.9 (5.0-6.7) |
| 2002 | 5.8 (5.5-6.2) | 5.5 (4.7-6.4) |
| 2003 | 6.0 (5.6-6.4) | 7.1 (6.1-8.0) |
| 2004 | 5.7 (5.3-6.1) | 6.1 (5.2-7.0) |
| 2005 | 5.7 (5.3-6.1) | 7.3 (6.4-8.3) |
| 2006 | 6.0 (5.6-6.4) | 6.8 (5.9-7.8) |
| 2007 | 6.2 (5.9-6.6) | 6.7 (5.8-7.6) |
| 2008 | 6.3 (5.9-6.7) | 8.7 (7.6-9.7) |
| 2009 | 6.7 (6.3-7.1) | 8.1 (7.1-9.1) |
| 2010 | 7.5 (7.1-7.9) | 8.6 (7.5-9.6) |
| 2011 | 7.6 (7.2-8.0) | 9.3 (8.2-10.4) |
| 2012 | 7.8 (7.4-8.2) | 9.2 (8.1-10.3) |
| 2013 | 7.8 (7.4-8.2) | 8.4 (7.3-9.4) |
| 2014 | 8.7 (8.2-9.1) | 11.2 (10.0-12.4) |
| 2015 | 8.9 (8.4-9.3) | 9.5 (8.4-10.6) |
| 2016 | 8.8 (8.3-9.2) | 11.8 (10.6-13.1) |
| 2017 | 9.0 (8.6-9.4) | 11.1 (9.9-12.3) |
| 2018 | 9.0 (8.6-9.4) | 12.4 (11.1-13.6) |
| 2019 | 9.7 (9.3-10.1) | 12.4 (11.1-13.7) |
| 2020 | 13.5 (12.9-14.0) | 16.7 (15.2-18.1) |
| **Overall** | 7.5 (7.4-7.6) | 8.9 (8.7-9.1) |

**Supplemental Table 9** Overall Individual disease–related Age-Adjusted Mortality Rates per 100,000 in the United States, 1999 to 2020

| **Age-Adjusted Rate (95% CI)** | | |
| --- | --- | --- |
| **Year** | **Pulmonary Embolism** | **Obesity** |
| 1999 | 14.2 (14.0-14.3) | 7.2 (7.1-7.3) |
| 2000 | 14.2 (14.0-14.4) | 7.5 (7.4-7.7) |
| 2001 | 14.4 (14.2-14.5) | 7.8 (7.6-7.9) |
| 2002 | 14.2 (14.1-14.4) | 8.9 (8.7-9.0) |
| 2003 | 14.4 (14.2-14.5) | 9.4 (9.3-9.6) |
| 2004 | 13.7 (13.6-13.9) | 9.8 (9.7-10.0) |
| 2005 | 13.9 (13.8-14.1) | 10.5 (10.4-10.7) |
| 2006 | 13.9 (13.7-14.0) | 10.8 (10.7-11.0) |
| 2007 | 13.6 (13.5-13.8) | 11.2 (11.0-11.3) |
| 2008 | 13.9 (13.7-14.0) | 11.7 (11.5-11.8) |
| 2009 | 13.4 (13.2-13.5) | 12.6 (12.5-12.8) |
| 2010 | 13.7 (13.5-13.8) | 13.0 (12.9-13.2) |
| 2011 | 13.8 (13.6-13.9) | 13.9 (13.8-14.1) |
| 2012 | 13.6 (13.5-13.8) | 14.5 (14.4-14.7) |
| 2013 | 13.5 (13.4-13.7) | 15.1 (15.0-15.3) |
| 2014 | 13.7 (13.5-13.8) | 16.1 (16.0-16.3) |
| 2015 | 13.9 (13.8-14.1) | 16.7 (16.5-16.9) |
| 2016 | 13.9 (13.8-14.1) | 17.4 (17.2-17.6) |
| 2017 | 14.3 (14.1-14.4) | 17.9 (17.7-18.1) |
| 2018 | 14.4 (14.2-14.5) | 18.4 (18.2-18.6) |
| 2019 | 14.5 (14.4-14.7) | 18.9 (18.8-19.1) |
| 2020 | 17.9 (17.7-18.1) | 29.5 (29.3-29.7) |
| **Overall** | 14.1 (14.1-14.2) | 14.0 (14.0-14.1) |

**Supplemental Table 10** Comparisons of the Trends in AAMR between Individual Disease (Pulmonary Embolism and Obesity alone) and Combined Pulmonary Embolism and Obesity-Related Mortality from 1999 to 2020 using Average Annual Percent Change (AAPC) and Average Annual Percent Change Difference (AAPCD)

| **Cohort or Comparison** | **Duration** | **AAPC (95% CI)** | **p-value of AAPC** | **AAPCD (95% CI)** | **p-value of AAPCD** |
| --- | --- | --- | --- | --- | --- |
| Pulmonary Embolism Alone | 1999-2020 | 0.40 (-0.04 to 0.85) | 0.074 | N/A | N/A |
| Pulmonary Embolism + Obesity | 1999-2020 | 4.02 (3.42 to 4.62) | <0.001 | N/A | N/A |
| Pulmonary Embolism + Obesity **Vs.** Pulmonary Embolism Alone | 1999-2020 | N/A | N/A | 3.61 (2.91 to 4.32) | <0.001 |
| Obesity Alone | 1999-2020 | 6.16 (5.13 to 7.20) | <0.001 | N/A | N/A |
| Pulmonary Embolism + Obesity **Vs.** Obesity Alone | 1999-2020 | N/A | N/A | -2.14 (-3.27 to -1.02) | <0.001 |
